# The Effect of Reagents Mimicking Oxidative Stress on Fibrinogen Function

**DOI:** 10.1155/2013/359621

**Published:** 2013-10-21

**Authors:** Jana Štikarová, Roman Kotlín, Tomáš Riedel, Jiří Suttnar, Kristýna Pimková, Leona Chrastinová, Jan E. Dyr

**Affiliations:** Institute of Hematology and Blood Transfusion, U Nemocnice 1, 128 00 Prague 2, Czech Republic

## Abstract

Fibrinogen is one of the plasma proteins most susceptible to oxidative modification. It has been suggested that modification of fibrinogen may cause thrombotic/bleeding complications associated with many pathophysiological states of organism. We exposed fibrinogen molecules to three different modification reagents—malondialdehyde, sodium hypochlorite, and peroxynitrite—that are presented to various degrees in different stages of oxidative stress. We studied the changes in fibrin network formation and platelet interactions with modified fibrinogens under flow conditions. The fastest modification of fibrinogen was caused by hypochlorite. Fibers from fibrinogen modified with either reagent were thinner in comparison with control fibers. We found that platelet dynamic adhesion was significantly lower on fibrinogen modified with malondialdehyde and significantly higher on fibrinogen modified either with hypochlorite or peroxynitrite reflecting different prothrombotic/antithrombotic properties of oxidatively modified fibrinogens. It seems that, in the complex reactions ongoing in living organisms at conditions of oxidation stress, hypochlorite modifies proteins (e.g., fibrinogen) faster and more preferentially than malondialdehyde. It suggests that the prothrombotic effects of prior fibrinogen modifications may outweigh the antithrombotic effect of malondialdehyde-modified fibrinogen in real living systems.

## 1. Introduction

The development of many pathological states and diseases is associated with activation and intensification of free-radical processes when reactive oxygen and nitrogen species (ROS, RNS, together abbreviated as RONS) are produced [1–3]. It was found that plasma fibrinogen was much more susceptible to oxidative modification compared to the other major plasma proteins, albumin, immunoglobulins, and transferrin [4].

Fibrinogen is an adhesive plasma protein, which plays a central role in haemostasis. It is a 340 kDa glycoprotein composed of three nonidentical peptide chains A*α*, B*β*, and *γ* connected with 29 disulfide bonds. Activation of the coagulation cascade converts soluble fibrinogen to insoluble fibrin, which produces together with platelets the haemostatic clot. Plasma fibrinogen is an important component of the coagulation cascade as well as a major determinant of blood viscosity and blood flow. Whereas the normal activation of the coagulation cascade is essential for life, inappropriate activation may result in thrombosis [5–7]. 

Modification of fibrinogen molecule affects haemostasis by changes in formation and architecture of fibrin network and by changes of fibrin(ogen) interactions with platelets, endothelial, and other cells via cell-membrane fibrin(ogen) receptors [8–15]. Fibrin(ogen) receptors can transduce intracellular signals upon fibrin(ogen) binding, whereas fibrin(ogen) binding proteins are either soluble or anchored molecules that bind fibrin(ogen) but have no documented ability to directly transduce intracellular signals upon fibrin(ogen) binding. The functional consequences of these protein-fibrin(ogen) interactions range from blood coagulation and initiation of angiogenesis to inflammation and propagation of infection.

Various methods are used to generate respective RONS for modeling the oxidative stress in *in vitro* systems. Thus, induced changes of proteins molecules mimic the results of oxidative and/or nitrative (patho)physiological reactions *in vivo*. 

In the present study, we selected malondialdehyde (MDA), sodium hypochlorite (NaOCl), and peroxynitrite (PN) as oxidative/nitrative reagents frequently used in protein chemistry. These reagents are (patho)physiologically present during oxidative stress. Fenton reaction (Haber-Weiss reaction) and myeloperoxidase production of hypochlorite are other *in vitro*-used systems that mimic oxidative stress.

MDA is an indicator of lipid peroxidation [16], and it is associated with a number of pathological processes, for example, atherosclerosis and inflammatory joint diseases. Reaction of MDA with proteins may result in inter-/intracross-linking of proteins or formation of carbonyl groups in proteins. 

Hypochlorous acid (HOCl) is produced physiologically during activation of phagocytes. Reaction of HOCl with proteins can result in the alteration of amino acid side chains, protein fragmentation [17], and cross-linking. HOCl-modified proteins were found in human atherosclerotic plaque tissue. Sodium hypochlorite simulates the reaction of HOCl with proteins [18].

Peroxynitrite formation was shown in chronic inflammation, and it is linked with development of atherosclerosis [8, 19]. Spontaneous reaction of PN with proteins leads to nitration of tyrosyls, oxidation of cysteinyl, methionyl, and tryptophanyl amino acid residues, formation of dityrosine and carbonyl groups, and protein fragmentation [19–21]. Peroxynitrite can be produced *in vitro* by decomposition of 3-morpholinosydnonimine (SIN-1) [2, 8].

The aim of the present study was to determine the influence of fibrinogen oxidative modifications (newly also including the effect of malondialdehyde) on platelet dynamic adhesion and fibrin network architecture. The study of the dynamic adhesiveness of platelets in the presence of oxidatively modified fibrinogen can help to assess the influence of platelet adhesion on the postischemic vessel wall.

## 2. Methods

### 2.1. Fibrinogen Modification

Lyophilized human fibrinogen (Sigma-Aldrich, Prague, Czech Republic) (4 mg/mL) was dissolved in phosphate buffer saline (PBS; 137 M NaCl, 2.7 mM KCl, 8 mM Na_2_HPO_4_·12H_2_O, 1.5 mM KH_2_PO_4_, and pH 7.4). Its concentration was determined spectrometrically at 278 nm using an extinction coefficient 15.1 for 10 mg/mL solution.

Fibrinogen was modified by three different systems: treatment of fibrinogen (a) by malondialdehyde (MDA; 10 mM; incubation times 30, 60, and 120 minutes; dark), (b) by sodium hypochlorite (NaOCl; 1.25 mM; incubation times 5, 10, and 20 minutes) [22], and (c) by 3-morpholinosydnonimine (SIN-1; 100 *μ*M; incubation times 30 and 60 minutes; vortexing every 10 min) [8]. All samples were incubated at 37°C. Control samples were exposed to conditions of modification but without modification species.

After incubation with modification species fibrinogen was purified by centrifugal gel filtration (Sephadex G-25 superfine; Pharmacia, Uppsala, Sweden). Protein concentration in eluate was estimated by Bradford protein assay.

### 2.2. Preparation of Modification Species

Malondialdehyde was prepared by acid hydrolysis of 1,1,3,3-tetramethoxypropane (Sigma-Aldrich, Prague, Czech Republic). MDA concentration was determined spectrometrically  (*ε*_245_ = 13  700 M^−1^ cm^−1^) [23].

Sodium hypochlorite (Sigma-Aldrich, Prague, Czech Republic) was prepared by dilution with PBS, pH 7.4. Concentration of NaOCl was determined spectrometrically (*ε*_290_ = 350 M^−1^ cm^−1^) [24].

3-Morpholinosydnonimine (Sigma-Aldrich, Prague, Czech Republic) was dissolved in 50 mM potassium phosphate buffer, pH 5.0 [8].

### 2.3. Carbonyl Quantification

Content of carbonyls was determined using essentially the method of Belisario et al. [12]. Briefly, control or oxidized fibrinogen was precipitated by trichloroacetic acid (TCA; Sigma-Aldrich,  Prague, Czech Republic) (10% w/v, final concentration) and consequently derivatized by 10 mM dinitrophenylhydrazine (DNPH; Sigma-Aldrich, Prague, Czech Republic). After the DNPH reaction, the protein was reprecipitated with ice-cold TCA (10% w/v, final concentration), and the pellet was washed three times with TCA (5%) and then with ethanol : ethyl acetate mixture (1 : 1). The protein pellet was dissolved in 6 M guanidine-HCl (Sigma-Aldrich, Prague, Czech Republic), and absorbance was monitored between 250 and 500 nm (Shimadzu UV-2401, Shimadzu Corp., Prague, Czech Republic). Extinction coefficients for the wavelengths 278 nm for hydrazone and fibrinogen are 9 460 l·mol^−1^·cm^−1^ and 57 000 l·mol^−1^·cm^−1^, respectively. Hydrazone extinction coefficient for 370 nm is 22 000 l·mol^−1^·cm^−1^ [12].

### 2.4. Fibrin Polymerization Curves Measurement

Thrombin-catalyzed fibrin polymerization was monitored at 350 nm in microplates at 37°C for 40 min (Synergy HT, BioTek AS, Prague, Czech Republic). 200 *μ*L of modified or control fibrinogen (1.25 mg/mL) in PBS was added to each well. Reaction was initiated by thrombin (EC 3.4.21.5; Sigma-Aldrich, Prague, Czech Republic) (50 *μ*L, final concentration 0.5 NIH U/mL). Samples were mixed automatically within instrument (5 s) immediately after the reaction start. Turbidity was measured every 20 s for 40 min [9].

### 2.5. Scanning Electron Microscopy

Fibrin networks were prepared in a polystyrene shallow well. Fibrinogen, modified and control, was mixed with thrombin (final concentration 2 NIH U/mL) and incubated in atmosphere of saturated water vapor pressure at room temperature for 3 hours. The networks were washed with PBS and water and subsequently dehydrated with a series of water-ethanol solutions with increasing ethanol concentration (0%, 25%, 50%, 75%, and 100%). Finally, the samples were dried using the CO_2_ critical point method (Balzers CPD 010) and coated with 4 nm thick platinum by sputtering (Balzers SCD 050). A TESCAN Vega Plus TS 5135 (Tescan, s.r.o., Brno, Czech Republic) electron microscope was used for the scanning observations. Images were evaluated using ImageJ data analysis software (http://rsbweb.nih.gov/ij/) [25].

### 2.6. Measurement of Platelet's Dynamic Adhesion by Cone and Plate Analyzer

Blood was drawn from healthy volunteers, who had not ingested any drug for at least two weeks, in accordance with the Ethical Committee regulations of our institute. 

Washed blood platelets were isolated by differential centrifugation of blood collected into ACD (citric acid/citrate/dextrose) solution 8.1 : 1.9 (v/v). Platelet rich plasma (PRP) was prepared from blood by centrifugation at 250 ×g at 37°C for 15 min. PRP containing prostaglandin E1 (1 *μ*M, final concentration; Sigma-Aldrich, Prague, Czech Republic) was incubated in a water bath at 37°C for 10 min and centrifuged at 1000 ×g at 37°C for 10 min. The resulting platelet pellet was resuspended in modified (Ca^2+^-free) Tyrode's buffer pH 6.2 (140 mM NaCl, 3 Mm KCl, 12 mM NaHCO_3_, 0.4 mM NaH_2_PO_4_·H_2_O, 2 mM MgCl_2_, and 5.55 mM glucose; pH was adjusted by H_3_PO_4_) in the presence of 1 *μ*M prostaglandin E1 and centrifuged at 600 ×g at 37°C for 10 min. The platelets were finally resuspended in a Tyrode's buffer pH 7.4 (140 mM NaCl, 3 mM KCl, 12 mM NaHCO_3_, 0.4 mM NaH_2_PO_4_·H_2_O, 1 mM MgCl_2_·6 H_2_O, 2 mM CaCl_2_·2 H_2_O and 5.55 mM glucose; pH was adjusted by Hepes) to a required concentration of platelets, equilibrated 30 min at 37°C and used for experiments within 1 h [26, 27]. 

Red blood cells were prepared from the same person's blood. Briefly, the whole blood was first centrifuged (250 ×g at 37°C for 15 min), and PRP was removed. The remaining blood sample was diluted in PBS (final volume of the whole blood sample) and centrifuged (220 ×g at 25°C for 10 min), and the supernatant was consequently removed. This step was repeated three times. The conditions of the last centrifugation were altered (2000 ×g at 25°C for 10 min), and red blood cells were resuspended in PBS [28].

After mixing a suspension of washed platelets and washed red blood cells (1 : 1) (final count of platelets and red blood cells was 200 000/*μ*L and 4 000 000/*μ*L, resp.), modified or control fibrinogen was added (final concentration 1 mg/mL). 

High shear was applied with a cone and plate analyzer, the Impact-R (DiaMed; Eurex Medica, Ostrava, Czech Republic) in accordance with manufacturer's manual. Sample (washed platelets, red blood cells, and fibrinogen) was placed onto a polystyrene plate onto which a Teflon cone was perfectly fitted. After incubation (10 s) shear was applied (shear rate 1800 s^−1^) for 2 minutes. Plates were then washed with deionized water and stained with May-Grűnwald (Merck, Prague, Czech Republic). 

Samples were analyzed using an image analyzing system that is a part of Cone and Platelet analyzer software. The images obtained by built-in camera were processed by the software that calculates surface coverage (SC), number (OB), and size of surface-bound objects (AS). Seven images were collected from each run, and the medians of the respective values were calculated by the analyzing system. The influence of fibrinogen modification on platelet adhesion was expressed as a percentage of surface coverage by adhered platelets in the presence of modified fibrinogen versus surface coverage by adhered platelets in the presence of control fibrinogen.

### 2.7. Statistical Analysis

Results are presented as mean ± SD and were performed in triplicate unless stated otherwise. The significance of differences was evaluated using Student's *t*-test. *P* values less than 0.05 (two-sided) were considered statistically significant.

## 3. Results

### 3.1. Carbonyl Quantification

**Figure 1:**
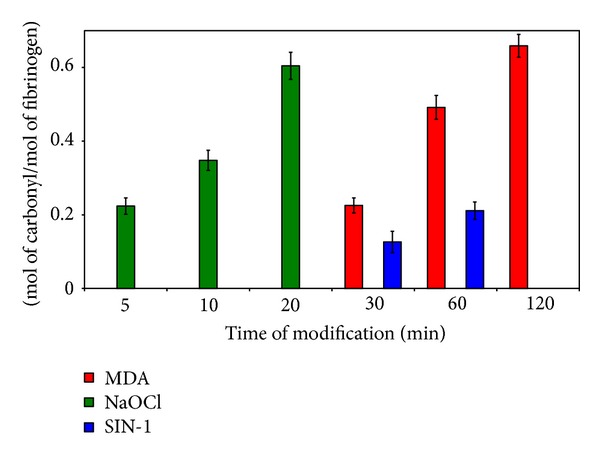
Evaluation of carbonyl groups in modified fibrinogen. Fibrinogen was treated with MDA, NaOCl, and SIN-1. Content of carbonyls was determined by DNPH derivatization [15]. Data are represented as mean ± SD from five independent experiments.

The ratio of carbonyl groups per fibrinogen molecule was counted. The amount of carbonyl groups in modified fibrinogen increased with time of modification in all three modification systems (Figure 1). Native fibrinogen contains approximately 0.03 mol carbonyl/mol fibrinogen. Carbonyl groups were also detected in control fibrinogens but their amount was significantly lower than in modified fibrinogens. These data suggest that the most effective modification was done by NaOCl (0.60 ± 0.04 mol carbonyl/mol fibrinogen). The highest content of carbonyl groups in molecule of fibrinogen was created by MDA modification (0.66 ± 0.03 mol carbonyl/mol fibrinogen), but longer reaction time was necessary. 

### 3.2. Fibrin Polymerization Curves Measurement

**Figure 2:**
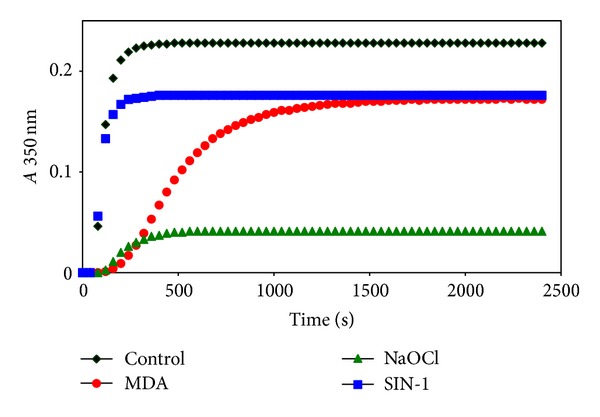
Representative curves of thrombin-catalyzed fibrin polymerization. Fibrin clot formation from modified (modification times: MDA 30 min, NaOCl 5 min, and SIN-1 30 min) or control fibrinogen (1 mg/mL) was monitored at 350 nm after the addition of thrombin (0.5 U/mL).

Polymerization of fibrin was monitored at 350 nm for 40 min (Figure 2).  Maximal absorption and reaction rate were calculated from five independent experiments and then the *t*-test was used. 

The significantly lower final optical density was obtained using NaOCl-modified fibrinogen as compared with control. The maximal velocity of fibrin network formation was also significantly lower as compared with control. We also found lower final optical density in polymerization of fibrin modified by MDA and SIN-1, but these differences did not reach significance (data not shown).

### 3.3. Scanning Electron Microscopy

**Figure 3:**
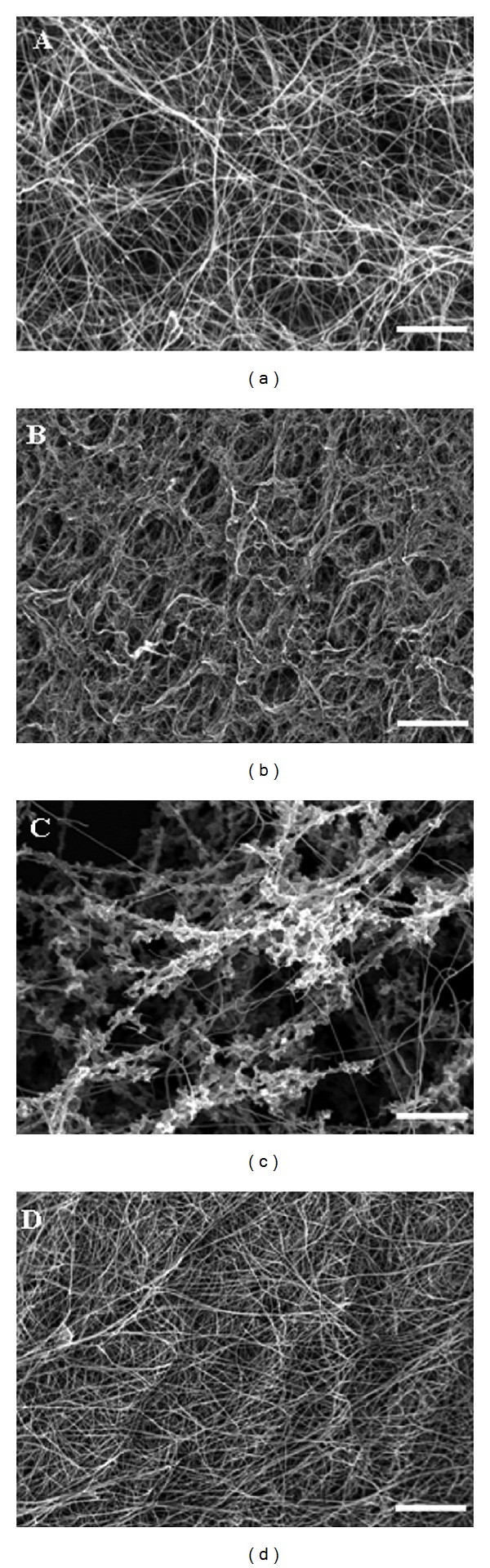
Representative SEM images of fibrin clots formed by modified and control fibrinogens. Control fibrinogen (a) and fibrinogen modified by (b) MDA, (c) NaOCl, and (d) SIN-1. Images were obtained from the clots formed by fibrinogen in the final time of modification (MDA 120 min, NaOCl 20 min, and SIN-1 60 min). The scale bar is 5 *μ*m.

The architecture of the fibrin clots was examined by scanning electron microscopy (SEM; Figure 3). Multiple images were taken throughout a fibrin net produced from fibrinogen modified by respective reagents. One of them mostly representing the modification with respective reagent was chosen for further examination. The significant changes between modified and control network architecture were found in clots from fibrinogen modified by all modification systems. Fibers from modified fibrinogen were significantly (*P* < 0.05) thinner in comparison with control fibers. The network from NaOCl treated fibrinogen was composed of thin fibers with bundles and with many pores, while clots formed by fibrin exposed to both SIN-1 and MDA were denser than control (Table 1).

### 3.4. Platelet's Dynamic Adhesion

**Figure 4:**
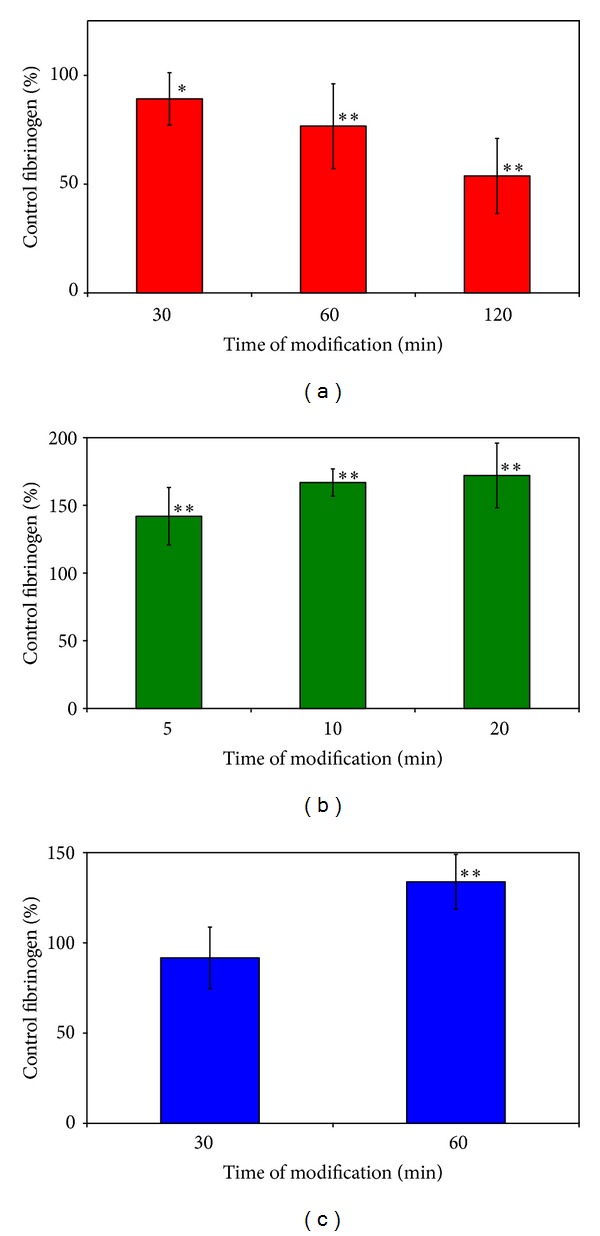
Dynamic adhesions (relative surface coverage) in the presence of control and modified fibrinogen. Fibrinogen (Fbg) modified by (a) MDA, (b) NaOCl, and (c) SIN-1. Data are expressed as a percentage of surface coverage in the presence of modified fibrinogens relatively to surface coverage in the presence of control fibrinogen. Results are represented as mean ± SD of nine values from three independent experiments (three values per experiment). Student's *t*-test probability: *P < 0.05; **P < 0.01.

Samples were analyzed with the image analyzing system. Platelet adhesion and aggregation were recorded by examination of the percentage of total area covered with platelets. All fibrinogen modification systems induced significant (*P* < 0.05) differences in platelet dynamic adhesion between modified and control fibrinogen. Fibrinogen modified by MDA greatly inhibited dynamic adhesion. This inhibition was increased with the time of modification (Figure 4(a)). The surface coverage in the presence of modified fibrinogen (120 min) reached 53.7 ± 17.3% of surface coverage of platelets in the presence of control fibrinogen. NaOCl treatment stimulated dynamic adhesion of platelets (Figure 4(b)). Modified fibrinogen stimulated dynamic adhesion to almost double values (172.0 ± 24.0%) in comparison with control fibrinogen. Significant increase was found between dynamic adhesion in the presence of modified fibrinogen by SIN-1 and control fibrinogen after 60 min of fibrinogen treatment (Figure 4(c); 133.8 ± 15.1%).

## 4. Discussion

The literary data of prothrombotic/antithrombotic (beneficial or harmful?) properties of oxidized fibrinogen are not straightforward. Vadseth et al. [8], Paton et al. [1], and Upchurch et al. [29] proposed prothrombotic state induced by oxidative posttranslational modification of fibrinogen while Shacter et al. [9] or Tetik et al.[13, 30] argued the opposite. 

Experimental conditions (concentration of added reagent and reactions time) were chosen to reflect real (patho)physiological (possible) situations in organism. MDA is a long-lived reagent, and its concentration in plasma from control is between 1 and 2 *μ*M [31]. Activation of platelets close to atherosclerotic plates, however, may lead to the production of a large amount of MDA, and thus, fibrin(ogen) located in these lesions could be exposed to extended modification [32]. Hypochlorite concentration was higher than that used in Vadseth et al. [8] (1.25 mM and 0.1 mM, resp.) but within limit set for real system. Concentration of hypochlorite in sites of inflammation might be 0.34 mM or greater [33]. Decomposition of SIN-1 leads to nitric oxide and superoxide production. These two molecules immediately form peroxynitrite (rate constant 10^10^ M^−1^ s^−1^) [8]. The concentration of peroxynitrite is estimated to be in nanomolar range concentration [34]. Peroxynitrite is produced by several cells as vascular endothelial cells or activated neutrophils, thus, fibrinogen in lesions could be exposed to a higher local concentration (up to 100 *μ*M) [35]. We used modification conditions as in Vadseth et al. [8].

All modification systems we employed initiated the increase of carbonyl groups content in fibrinogen. The highest content of carbonyl groups was reached using MDA, but the modification induced by NaOCl proceeded more rapidly. The range of fibrinogen carbonylation was within limit values found in posterior myocardial infarct patients, thus reflecting the real fibrinogen modifications in cardiovascular diseases [1].

The singlet oxygen (^l^O_2_) is formed from hypochlorite either in the presence of hydrogen peroxide or upon its simple acidification onset at pH 8 [36] and plays a significant role in oxidative stress. ^l^O_2_ production by the spontaneous dismutation of O_2_^∙−^ does have also physiological relevance. Singlet oxygen then inactivates fibrinogen, factor V, factor VIII, factor X, and platelet aggregation of human blood [37]. Moreover, thrombin converts oxidized fibrinogen into a soluble stimulator of tissue-type plasminogen activator [38]. Changes in functional activities of plasma fibrinogen after treatment with methylene blue and red light *in vitro*, where singlet oxygen is formed, were observed by Suontaka et al.[39].

Lupidi states that also peroxynitrite-mediated oxidation of fibrinogen inhibits clot formation [21]. However, it was proved that peroxynitrite does not decompose to singlet oxygen and nitroxyl (NO_2_) [40].

Taking all these facts together, the singlet oxygen plays a complex role in blood haemostasis, modifies many coagulation factors, and affects platelet aggregation. The oxidative modification of fibrinogen with reagents used in our study could be also partly caused by singlet oxygen, since at pH 7.4, we used in our experiments, the hypochlorite decomposes partly to it. Neither peroxynitrite nor malondialdehyde produces singlet oxygen; however, they modify fibrinogen and other proteins by different mechanisms. The platelet interaction with modified fibrinogen was studied at conditions, where modification reagents were entirely removed and thus their influence on platelets was excluded.

We found that fibers from fibrinogen modified with all used reagents were markedly thinner in comparison with control fibers. The finding was supported by measurements of modified fibrin polymerization curves that showed lower turbidity as compared with control samples. Only fibrin clot made from fibrinogen modified with hypochlorite was composed of large bundles of thin fibrin fibers.

In a previous work, we studied the thrombin kinetics of fibrinopeptides release from fibrinogen modified with the same set of reagents and found significantly decreased rate of both fibrinopeptides A and B release as compared with control sample [41]. It is well known that fibrinopeptide B cleavage occurs primarily from fibrin oligomers and fibrinogen/fibrin complexes [42] and enhances lateral aggregation. It seems that fibrin oligomer formation from modified fibrinogen molecules is hindered due to reagents action and therefore the resulting clots are made up of thinner fibers as compared with control samples. 

We have found significantly decreased platelet adhesion on fibrinogen modified with MDA and significantly higher platelet adhesion on fibrinogen modified either with hypochlorite or with SIN-1. The variety of obtained adhesion results reflects differences in published prothrombotic/antithrombotic properties of oxidatively modified fibrinogen [1, 8, 9, 29] and suggests that the respective reagents differ in their effect on fibrin(ogen) properties. Reaction of fibrinogen with malondialdehyde results mainly in modification of *ε*-amino groups of lysyl residues. The cross-linking of fibrinogen by bifunctional malondialdehyde can involve not only reaction with monomer MDA molecules, but also reaction of MDA oligomers [43]. On the other hand, hypochlorite reactions with proteins form wide spectra of compounds: lysine chloramines from lysine residues, oxidation products of cystine/cysteine residues, methionine residues, tryptophan residues, and production of chlorotyrosine and dityrosine; fragmentation of proteins can also take place [44]. N-Chloramine derivatives can subsequently break down to form reactive aldehydes that can cause cross-linking of even partially fragmented fibrinogen. Therefore, the modification of fibrinogen with hypochlorite is much more complex as compared with MDA and due to nonspecific charge and hydrophobicity effects can enhance platelet adhesion. Importantly, the reaction of fibrinogen with hypochlorite was in our experiments much faster as compared with malondialdehyde. It seems that, in the complex reactions ongoing in living organisms at conditions of oxidation stress, the strong reagents (e.g., hypochlorite formed by myeloperoxidase) modify proteins (e.g. fibrinogen) faster and preferentially as compared with weaker reagents (e.g. MDA). Thus, the prothrombotic effects of prior fibrinogen modifications can make the antithrombotic effect of malondialdehyde-modified fibrinogen in real living systems prevail. Furthermore, MDA and other reactive aldehydes are formed as a consequence of lipid peroxidation mediated with hydroxyl radical formed, for example, by Haber-Weiss reaction. The ROS, like superoxide, peroxynitrite, and hypochlorite (strong reagents), have short half-life time and react immediately after their formation in the site of oxidative stress (e.g. inflammation) with proteins. In contrast, the aldehydes covalently modify proteins localized throughout the cell at a later time and relatively far away from the initial site of primary ROS formation. The observed decreased and slower production of fibrin network from modified fibrinogen molecules seems to cause antithrombotic effects as at first proposed by Shacter et al. [9]. Nevertheless, the changes in fibrin network properties produced by respective reagents can either support or weaken fibrin(ogen) interaction with platelets. Resulting effects not only depend on the changes in fibrinogen molecule but also vary with flow conditions in the experiment. Our experiments with SIN-1 modification of fibrinogen revealed that, at physiological shear rate (1800 s^−1^), the relatively small extent of carbonylation (as compared with both other reagents) leads to the significant enhancement of dynamic platelet adhesion as compared with control sample. Thus, at physiological shear stress conditions, the SIN-1 fibrinogen modification had prothrombotic potential—in agreement with the results of Vadseth et al. [8].

As these experiments were performed in a matrix different from plasma, the results may not correspond to the *in vivo* conditions. Pieters et al. [45] stated in their work that plasma may buffer the negative effect of oxidative stress on fibrinogen molecule. Nevertheless, extended changes in the structure of fibrinogen molecule might be far beyond the plasma buffer capacity.

In conclusion, the oxidative modification of fibrinogen molecule has significant influence on its properties and depends on the intensity and time of the oxidative stress. Since the oxidative modification of proteins including fibrinogen depends on a complex of oxidative stress reactions, the *in vitro* modification with respective reagents is only an approximation of the real conditions. However, our results clearly stress the importance of oxidative fibrinogen changes in thrombotic episodes. 

## Figures and Tables

**Table 1:** Fibers thickness and average number of fibrin fibers per 1 *μ*m^2 ^of fibrin clot.

	MDA		NaOCl		SIN-1	
	Control fibrinogen	Modified fibrinogen	Control fibrinogen	Modified fibrinogen	Control fibrinogen	Modified fibrinogen
Fibers thickness (nm)	115.2 ± 30.8	102.0 ± 21.8	136.7 ± 48.8	107.4 ± 23.7	130.3 ± 43.0	102.1 ± 34.0
Average no. of fibers strands per field (1 *μ*m^2^)	12.9 ± 1.0	20.5 ± 3.3	13.1 ± 1.8	2.0 ± 1.2	14.1 ± 1.1	20.4 ± 2.2

Quantity and thickness of fibrin fibers were obtained from three experiments. Thickness of fibers is presented as mean of 45 values (15 values per experiment) with SD. Count of fibers was acquired as mean value from 6 areas (2 areas per experiment). Differences between control and modified fibrinogen were significant in both thickness and quantity parameters (paired Student's *t*-test) with probability *P* < 0.05.

## Abbreviations

ROS: Reactive oxygen species
RNS: Reactive nitrogen species
RONS: Reactive oxygen and nitrogen species
MDA: Malondialdehyde
NaOCl: Sodium hypochlorite
HOCl: Hypochlorous acid
PN: Peroxynitrite
SIN-1: 3-Morpholinosydnonimine
PBS: Phosphate buffer saline
TCA: Trichloroacetic acid
DNPH: Dinitrophenylhydrazine
ACD: Citric acid/citrate/dextrose
PRP: Platelet rich plasma
SEM: Scanning electron microscopy.
